# Adherence to the dietary index for gut microbiota and the 5-year incidence of metabolic dysfunction-associated steatotic liver disease in Iranian adults: a prospective cohort study

**DOI:** 10.1186/s12876-026-05036-5

**Published:** 2026-06-20

**Authors:** Ali Nikparast, Matin Sepehrinia, Nazanin Zamanian, Jalaledin Mirzay Razaz, Ali Tabatabaeyan, Saeid Hadi, Reza Homayounfar

**Affiliations:** 1 Student Research Committee, Tabriz University of Medical Sciences, Tabriz, Iran; 2 National Nutrition and Food Technology Research Institute (WHO Collaborating Center), Faculty of Nutrition Sciences and Food Technology, Shahid Beheshti University of Medical Sciences, P.O. 19395- 4741, Tehran, Iran; 3 Students’ Scientific Research Center (SSRC), Tehran University of Medical Sciences, Tehran, Iran; 4 Department of Clinical Nutrition, School of Nutritional Sciences and Dietetics, Tehran University of Medical Sciences, Tehran, Iran; 5 Nutrition and Health Research Center, Shahid Beheshti University of Medical Sciences, Tehran, Iran; 6 Department of Community Nutrition, Faculty of Nutrition and Food Technology, National Nutrition and Food Technology Research Institute, Shahid Beheshti University of Medical Sciences, Tehran, Iran; 7 Department of Clinical Nutrition, School of Nutrition and Food Science, Nutrition and Food Security Research Center, Isfahan University of Medical Sciences, Isfahan, Iran; 8 Nutrition and Food Health Research Center, AJA University of Medical Sciences, Tehran, Iran

**Keywords:** Gut microbiome, Hepatic steatosis, Nutrition, Gut-liver axis, Dietary patterns

## Abstract

**Background:**

Diet is a key modulator of gut microbiota and may influence the development of metabolic dysfunction-associated steatotic liver disease (MASLD). The Dietary Index for Gut Microbiota (DI-GM) has been proposed to capture the overall capacity of diet to promote a favorable gut microbial profile. Prospective evidence linking DI-GM to MASLD risk remains limited.

**Methods:**

This prospective analysis included 5,058 adults without MASLD at baseline from the Monitoring of Metabolic Diseases Risk Factors in Tehran (MMRT) study. Dietary intake was assessed using a validated 125-item food frequency questionnaire. The five-year incidence of MASLD was evaluated using multivariable logistic regression models, and associations were expressed as odds ratios (ORs) with 95% confidence intervals (CIs). Subgroup analyses were conducted to assess potential effect modification. Sensitivity analyses examined the robustness of results after excluding participants with substantial weight gain and after additional adjustment for metabolic and dietary factors. Mediation analyses were performed to explore potential pathways underlying the observed associations.

**Results:**

Over five years of follow-up, 562 participants developed MASLD. Higher DI-GM scores were associated with a lower likelihood of incident MASLD. In the fully adjusted model, individuals in the highest quartile of DI-GM had 42% lower odds of MASLD compared with those in the lowest quartile (OR:0.58; 95%CI:0.42–0.80; P-trend < 0.01). Each one-standard-deviation increment in DI-GM score was associated with reduced odds of MASLD (OR:0.72; 95%CI:0.65–0.81;P-value < 0.001). The inverse association was more pronounced among women and participants aged ≥ 45 years (P-interaction < 0.01). Mediation analyses suggested that CAP, HOMA-IR, and serum vitamin D partially explained the association.

**Conclusions:**

Greater adherence to a diet supportive of gut microbiota, as reflected by higher DI-GM scores, was associated with a lower five-year risk of MASLD. These findings highlight the potential role of microbiota-related dietary patterns in MASLD prevention.

**Supplementary Information:**

The online version contains supplementary material available at https://doi.org/10.1186/s12876-026-05036-5.

## Background

Metabolic dysfunction-associated steatosis liver disease (MASLD) is a recently endorsed term that reflects the complex interplay between hepatic steatosis and systemic metabolic dysfunction [1]. It is characterized by excessive hepatic fat accumulation in the presence of one or more cardiometabolic risk factors [1]. MASLD has emerged as a major global health concern, affecting nearly 38% of the adult population worldwide, largely driven by the increasing prevalence of obesity, insulin resistance, and metabolic syndrome [2, 3]. Beyond liver-related complications, MASLD is strongly associated with an elevated risk of cardiovascular disease, type 2 diabetes mellitus, chronic kidney disease, and certain cancers, contributing substantially to global morbidity and mortality [1–4].

The pathogenesis of MASLD is multifactorial and involves a complex interaction of genetic, metabolic, and environmental factors [4]. In recent years, growing evidence has highlighted the critical role of the gut–liver axis in the development and progression of MASLD [5]. This bidirectional communication system enables the transport of microbial metabolites and endotoxins from the gut to the liver via the portal circulation, thereby influencing hepatic metabolism and immune responses [6]. Dysbiosis of the gut microbiota has been linked to increased intestinal permeability, systemic inflammation, insulin resistance, and alterations in bile acid metabolism, all of which contribute to hepatic lipid accumulation and disease progression [6, 7].

Diet is a key modifiable determinant of gut microbiota composition and function and may therefore play a pivotal role in MASLD prevention [4, 8]. To better capture the relationship between diet and gut microbial health, Kase et al. developed the Dietary Index for Gut Microbiota (DI-GM) based on a comprehensive synthesis of 106 studies examining diet–microbiota interactions [9]. Emerging evidence from cross-sectional studies suggests that higher adherence to DI-GM is associated with a lower risk of non-alcoholic fatty liver disease (NAFLD) and MASLD, as well as improved metabolic profiles [10–14]. However, existing studies are predominantly cross-sectional, limiting causal inference, and have largely been conducted in Western populations. Therefore, the generalizability of these findings to other populations with distinct dietary patterns and metabolic characteristics remains uncertain.

To address these gaps, the present prospective cohort study aimed to investigate the association between the DI-GM and the five-year incidence of MASLD in a large sample of Iranian adults.

## Methods

### Study population

This prospective cohort study was conducted using data from the Monitoring of Metabolic Diseases Risk Factors in Tehran (MMRT) study designed to investigate risk factors for non-communicable diseases among adults in Tehran, Iran [15]. Participants were recruited through convenience sampling from individuals aged 20 to 70 years who visited a comprehensive health center. Between January 2017 and December 2018, a total of 8,760 individuals were approached, of whom 7,836 consented to participate (response rate: 89.4%). All participants provided written informed consent and were assigned anonymized identification codes to protect confidentiality.

Baseline data were collected through structured face-to-face interviews, clinical assessments, and laboratory measurements, encompassing demographic characteristics, lifestyle behaviors, medical history, dietary intake, and anthropometric indicators. A follow-up assessment was conducted five years later (January 2022–December 2023), during which all initial participants were invited to undergo repeat evaluations using protocols identical to baseline procedures. The follow-up phase achieved a high participation rate (95%), with 7,600 individuals completing the reassessment.

**Fig. 1:**
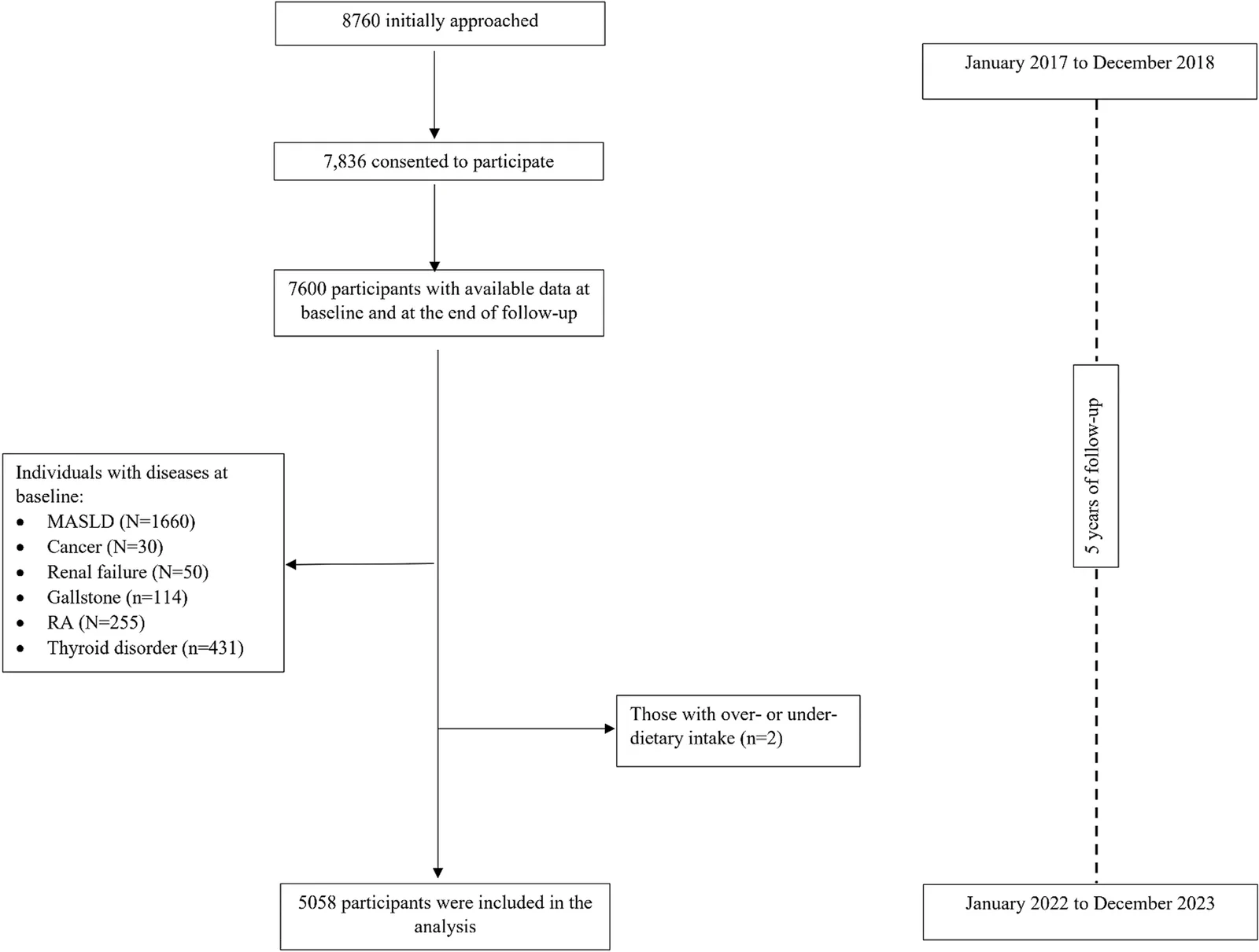
STROBE flow chart of participant selection and follow-up

To construct the analytical sample, individuals with pre-existing MASLD (*n* = 1,660), cancer (*n* = 30), renal failure (*n* = 50), gallstones (*n* = 114), rheumatoid arthritis (*n* = 255), thyroid disorders (*n* = 431), and implausible dietary energy intakes (defined as total energy intake values beyond ± 3 standard deviations from the mean daily energy intake of the study population) (*n* = 2) were excluded. The final study population consisted of 5,058 participants for whom all clinical, biochemical, and dietary variables required for analysis were fully available at both time points; no item-level missing data were present, and no imputation was performed. The detailed participant selection process is illustrated in Fig. 1. No formal a priori sample size calculation was performed because this study was based on an existing prospective cohort. The analytical sample consisted of all eligible participants from the MMRT cohort who met the predefined inclusion and exclusion criteria and had complete baseline and follow-up data. The study protocol received ethical approval from the ethics committee of National Nutrition and Food Technology Research Institute (Ethics code: IR.SBMU.NNFTRI.REC.1404.003) and adhered to the principles of the Declaration of Helsinki. Participants who were lost to follow-up were compared with those who remained in the study in terms of baseline characteristics to assess potential selection bias. This study was designed and reported in accordance with the Strengthening the Reporting of Observational Studies in Epidemiology (STROBE) guidelines to ensure transparent and complete reporting of observational research.

### Assessment of hepatic steatosis and MASLD

Liver steatosis was assessed using transient elastography (FibroScan™, Echosens, Paris, France) at both baseline (2017–2018) and the five-year follow-up (2022–2023). This allowed for the evaluation of changes in liver fat status over time and the identification of incident MASLD cases. The Controlled Attenuation Parameter (CAP), measured in decibels per meter (dB/m), was used to quantify hepatic fat content. A CAP value ≥ 238 dB/m was indicative of hepatic steatosis [16]. MASLD was diagnosed based on the presence of hepatic steatosis in combination with at least one metabolic risk factor: elevated BMI (≥ 25 kg/m²) or waist circumference (> 94 cm for men, > 80 cm for women), hypertension (≥ 130/85 mmHg or antihypertensive medication), hypertriglyceridemia (≥ 150 mg/dL or lipid-lowering therapy), low HDL-C (< 40 mg/dL in men, < 50 mg/dL in women or therapy), impaired fasting glucose (100–125 mg/dL), elevated HbA1c (5.7–6.4%), 2-hour postprandial glucose ≥ 140 mg/dL, or diagnosed diabetes mellitus or anti-diabetic treatment [1].

### Dietary assessment and DI-GM calculation

Dietary intake was evaluated using a validated 125-item Food Frequency Questionnaire (FFQ), adapted from the Willett format and tailored to reflect typical Iranian dietary habits [17]. This instrument has been previously validated among Iranian adults in the PERSIAN Cohort Study. Participants reported their frequency of consumption over the past year for each food item, choosing from daily, weekly, monthly, or yearly options. Standard serving sizes were defined based on the U.S. Department of Agriculture (USDA) food database, with household measures converted to gram equivalents when necessary. Nutrient and food composition data were primarily sourced from the USDA Food Composition Table, supplemented by the Iranian Food Composition Table for local items.

The DI-GM was calculated based on available dietary components in our dataset, following the framework proposed by Kase et al. [18]. Due to the unavailability of two items (avocado and green tea), a primary DI-GM score was constructed using 12 components. These included eight components considered beneficial for gut microbial diversity (fermented dairy products, chickpeas, soybeans, whole grains, dietary fiber, cranberries, broccoli, and coffee) and four components considered detrimental (refined grains, red meat, processed meats, and diets in which fat contributes ≥ 40% of total energy intake). For each beneficial component, participants received a score of 1 if their intake was above the sex-specific median; for detrimental components, a score of 1 was assigned if intake was below the median. The use of sex-specific medians for scoring was consistent with the original DI-GM methodology and allowed for population-specific dietary distribution. The total score ranged from 0 to 12, with higher scores indicating greater adherence to a gut microbiota–friendly dietary pattern. To evaluate the robustness of our findings and facilitate comparison with previous studies, we performed an additional sensitivity analysis by constructing an alternative 14-item DI-GM score under prespecified assumptions regarding avocado and green tea consumption, as these items were not captured by the study FFQ and therefore could not be directly measured. Results based on this alternative score were considered exploratory and were interpreted cautiously.

### Non-dietary assessment

Sociodemographic data, and health behaviors, were obtained through a standardized, interviewer-administered questionnaire. Variables included age, sex, education level, smoking status, medication use, and comorbidities. Socioeconomic status was evaluated using a wealth score index (WSI) derived from Multiple Correspondence Analysis (MCA), based on ownership of household appliances, digital devices, transportation means, type of television, and history of international travel [19]. Physical activity was assessed using the Modifiable Activity Questionnaire (MAQ), which calculates weekly metabolic equivalent task (MET) hours. The MAQ has been validated for use in Iranian populations [20].

### Anthropometric and biochemical measurements

Trained personnel measured weight, height, and waist circumference using standardized protocols. Body mass index (BMI) was calculated as weight (kg) divided by height squared (m²). Body composition was assessed via dual-energy X-ray absorptiometry (DXA; Lunar iDXA, GE Healthcare, USA), and fat mass index (FMI) was calculated by dividing total fat mass by height squared [21].

Blood pressure was measured using an automated sphygmomanometer after the participant was seated at rest for five minutes. The average of two measurements was used for analysis.

Fasting venous blood samples were collected after an overnight fast lasting between 8 and 12 h. The serum levels of fasting blood sugar (FBS), triglycerides (TG), total cholesterol (TC), and high-density lipoprotein cholesterol (HDL-C) were analyzed using enzymatic colorimetric methods with an automated analyzer. All measurements were conducted using the AutoAnalyzer system (Selectra E, Vitalab, Holliston, the Netherlands) in conjunction with Pars Azmoon kits. The assessment of low-density lipoprotein cholesterol (LDL-C) levels was conducted using the Friedewald formula [22]. Insulin levels were measured using electrochemiluminescence immunoassay, and insulin resistance was estimated via HOMA-IR: (Fasting blood sugar (mg/dl)*Fasting insulin (µU/mL))/405. Liver enzymes, including alanine aminotransferase (ALT), aspartate aminotransferase (AST), and gamma-glutamyl transferase (GGT), were assessed using standard enzymatic assays. Serum levels of C-reactive protein (CRP), vitamin D, and uric acid were also measured using standard laboratory methods.

### Statistical analysis

Descriptive statistics were used to characterize participants across quartiles of the DI-GM, with continuous variables expressed as means ± standard deviations and categorical variables as counts and percentages. Differences across quartiles were assessed using linear regression for continuous variables and chi-square (χ²) tests for categorical variables. In the primary analysis, DI-GM scores were categorized into true population-based quartiles, with quartile boundaries determined empirically from the distribution of scores within our study cohort to ensure approximately equal participant allocation across groups. This approach represents a methodological refinement from prior NHANES-based investigations of DI-GM, which employed fixed score-based thresholds (e.g., scores 0–3, 4, 5, and ≥ 6) reflecting the discrete and bounded nature of the index [11–14]. While such cutpoints carry interpretive value in populations where DI-GM scores follow a similar distribution, their application in our cohort resulted in substantially unequal group sizes—a limitation that could compromise the precision and comparability of effect estimates across categories. Accordingly, true quartile categorization was adopted to optimize statistical efficiency and ensure methodologically rigorous between-group comparisons. Associations between DI-GM and the odds of MASLD were assessed using multivariable logistic regression models. Because the exact timing of MASLD onset during follow-up was unavailable and all participants underwent outcome assessment at approximately the same five-year interval, time-to-event analyses such as Cox proportional hazards regression could not be appropriately implemented. Therefore, logistic regression was considered the most suitable approach for modeling incident MASLD and estimating odds ratios (ORs). ORs and 95% confidence intervals (CIs) were reported for each quartile of DI-GM, with the lowest quartile serving as the reference group. The first model was adjusted for age and sex. The second model included additional adjustments for FMI, PA, WSI, education level, smoking status, sleep disorders, T2DM, and hypertension. The third model was further adjusted for total energy intake. Candidate covariates were selected a priori based on previous literature, biological plausibility, and their established associations with both dietary patterns and MASLD risk [9, 23, 24]. These variables were retained in multivariable models irrespective of their statistical significance in univariate analyses. Trend analyses were conducted by assigning the median value of each quartile and modeling it as a continuous variable. In addition, DI-GM was analyzed as a continuous variable (per standard deviation increase). To assess the robustness of the findings and address potential bias arising from the modified 12-item index, all regression analyses were repeated using an alternative 14-item DI-GM score constructed under prespecified assumptions regarding avocado and green tea consumption.

Subgroup variables were categorized using predefined cut-off points. Age (< 45 and ≥ 45 years) and BMI (< 25 and ≥ 25 kg/m²) were classified according to commonly used clinical thresholds in previous studies. Wealth score index and physical activity were dichotomized based on their median values in the study population. Smoking status was categorized as yes/no. Hypertension was defined according to the Joint National Committee 7 (JNC-7) criteria (systolic blood pressure ≥ 140 mmHg, diastolic blood pressure ≥ 90 mmHg, or current use of antihypertensive medications), and type 2 diabetes mellitus was defined based on the American Diabetes Association (ADA) diagnostic criteria (fasting plasma glucose ≥ 126 mg/dL, physician diagnosis of diabetes, or use of glucose-lowering medications) [25, 26]. A non-linear dose-response association between DI-GM and MASLD odds was modeled using random-effects restricted cubic splines with three knots strategically placed at the 10th, 50th, and 90th percentiles of the DI-GM distribution [27].

Sensitivity analyses were performed to assess the robustness of the observed associations. These included: (1) exclusion of participants with > 10% weight gain from baseline; and (2) additional adjustment of the fully adjusted model for selected baseline metabolic and dietary variables, including CAP, HOMA-IR, triglyceride-to-HDL ratio, CRP, serum vitamin D, uric acid, dietary saturated fatty acids, total dietary fiber, and dietary sugar. These variables were selected based on their potential roles as metabolic intermediates or dietary confounders in the relationship between DI-GM and MASLD. CAP and HOMA-IR were additionally evaluated as potential intermediate variables because hepatic steatosis and insulin resistance represent key biological pathways linking dietary patterns to MASLD. However, because hepatic steatosis assessed by CAP forms part of the diagnostic framework for MASLD, analyses involving CAP were considered exploratory and were interpreted cautiously.

Mediation analyses were performed using the “mediation” package in R to evaluate the potential mediating roles of metabolic biomarkers. Direct, indirect, and total effects were estimated using a causal mediation analysis framework with 10,000 bootstrap replications to obtain robust confidence intervals. The proportion of the total effect mediated by each mediator was also calculated to quantify the extent to which these variables contributed to the observed association between the dietary index for gut microbiota and MASLD. The mediation analyses were exploratory and intended to evaluate potential biological pathways rather than establish causal mediation effects. In particular, the interpretation of CAP as a mediator warrants caution because hepatic steatosis, as assessed by CAP, is closely related to the outcome definition of MASLD. Furthermore, the interpretation of the mediation analyses relies on several assumptions, including the absence of unmeasured confounding between exposure, mediator, and outcome, as well as correct temporal ordering of these variables. Because the mediators were measured at baseline and the exact timing of disease onset was unavailable, these assumptions may not be fully satisfied and the findings should therefore be interpreted cautiously. All statistical analyses were conducted using Stata version 17 (StataCorp, College Station, TX, USA) and R software version 4.5.0 (R Foundation for Statistical Computing, Vienna, Austria). A two-sided p-value less than 0.05 was considered indicative of statistical significance.

Several strategies were employed to minimize potential sources of bias, including the use of validated questionnaires, standardized measurement protocols, exclusion criteria, and adjustment for potential confounders.

## Results

### Baseline characteristics of participants

A total of 5,058 participants were included in the analysis. The baseline characteristics of participants across quartiles of the DI-GM are presented in Table 1. Participants in the highest quartile (Q4) were significantly younger and more likely to be male compared with those in the lowest quartile (Q1) (P-trend < 0.001 for both). Physical activity levels and WSI increased progressively across quartiles of DI-GM (both P-trend < 0.001). No significant differences were observed for smoking status or educational level.

**Table 1:** Baseline characteristics of participants across quartiles of dietary index for gut microbiota^a^

Characteristics	Total study population (analytical sample) (*n* = 5058)	Q1 (*n* = 1264)	Q2 (*n* = 1264)	Q3 (*n* = 1262)	Q4 (*n* = 1266)	*P*-trend^b^
Age	41.95 ± 9.90	42.98 ± 10.08	42.25 ± 9.59	41.39 ± 9.70	41.18 ± 9.75	**< 0.001**
Men, n (%)	2665 (52.7%)	551 (43.6)	608 (48.1)	755 (59.7)	751 (59.3)	**< 0.001**
Smoker, n (%)	599 (11.9%)	132 (10.4)	153 (12.1)	148 (11.7)	166 (13.1)	0.22
Education, n (%)						
Low	928 (18.3%)	220 (17.4)	235 (18.6)	248 (19.6)	225 (17.8)	0.51
Middle	2984 (59.0%)	734 (59.1)	752 (59.5)	743 (58.5)	755 (59.6)	
High	1146 (22.7%)	310 (24.5)	277 (21.9)	273 (21.6)	286 (22.6)	
WSI	71.41 ± 14.28	69.62 ± 13.97	71.04 ± 14.84	72.61 ± 14.08	72.36 ± 14.00	**< 0.001**
Physical activity (MET/H/week)	24.01 ± 2.75	23.32 ± 2.45	23.94 ± 2.73	24.36 ± 2.89	24.39 ± 2.75	**< 0.001**
Weight (kg)	84.90 ± 13.47	88.53 ± 13.95	85.52 ± 13.85	83.20 ± 12.73	82.32 ± 12.40	**< 0.001**
Height (cm)	166.74 ± 6.14	166.22 ± 6.07	166.41 ± 6.16	167.18 ± 6.12	167.14 ± 6.13	**< 0.001**
Waist circumference (cm)	84.82 ± 14.53	89.08 ± 15.91	85.67 ± 15.04	82.63 ± 13.67	81.86 ± 12.09	**< 0.001**
Body mass index (kg/m^2^)	27.31 ± 4.51	28.38 ± 4.50	27.46 ± 4.49	26.83 ± 4.41	26.54 ± 4.39	**< 0.001**
Fat mass index (kg/m^2^)	7.28 ± 3.22	8.45 ± 3.49	7.54 ± 3.30	6.74 ± 2.94	6.40 ± 2.68	**< 0.001**
SBP (mmHg)	118.33 ± 7.85	118.90 ± 8.02	118.68 ± 7.97	118.04 ± 7.75	117.60 ± 7.59	**< 0.001**
DBP (mmHg)	72.64 ± 9.22	73.68 ± 4.35	72.74 ± 9.09	72.21 ± 9.25	71.90 ± 9.08	**< 0.001**
Fasting blood sugar (mg/dl)	97.92 ± 14.69	100.37 ± 14.59	98.00 ± 14.42	97.04 ± 14.83	96.26 ± 14.58	**< 0.001**
Fasting Insulin (µU/mL)	8.42 ± 2.73	9.54 ± 2.93	8.60 ± 2.82	7.92 ± 2.49	7.60 ± 2.17	**< 0.001**
HOMA-IR	1.29 ± 0.54	1.55 ± 0.55	1.33 ± 0.55	1.17 ± 0.48	1.07 ± 0.40	**< 0.001**
Triglyceride (mg/dl)	138.31 ± 16.69	142.15 ± 17.57	140.05 ± 17.39	136.04 ± 18.55	135.00 ± 14.73	**< 0.001**
Total cholesterol (mg/dl)	170.52 ± 26.73	175.25 ± 29.48	170.32 ± 26.95	169.38 ± 25.64	167.12 ± 23.88	**< 0.001**
LDL-C (mg/dl)	95.00 ± 15.24	97.09 ± 15.31	95.33 ± 15.45	94.24 ± 15.21	93.31 ± 14.74	**< 0.001**
HDL-C (mg/dl)	44.45 ± 7.10	42.67 ± 6.70	44.25 ± 7.20	45.20 ± 6.99	45.66 ± 7.13	**< 0.001**
ALT (U/L)	26.86 ± 4.66	27.33 ± 4.83	26.73 ± 4.73	26.85 ± 4.64	26.52 ± 4.35	**< 0.001**
AST (U/L)	24.43 ± 4.91	25.06 ± 4.99	24.43 ± 4.93	24.38 ± 4.93	23.84 ± 4.71	**< 0.001**
GGT (U/L)	24.80 ± 8.44	29.42 ± 8.22	25.62 ± 8.50	22.99 ± 7.79	21.17 ± 6.78	**< 0.001**
CAP (dB/m)	175.34 ± 36.17	193.71 ± 34.81	177.52 ± 36.37	168.31 ± 34.41	161.83 ± 30.73	**< 0.001**
CRP (mg/dl)	1.87 ± 1.66	2.63 ± 1.99	1.93 ± 1.70	1.58 ± 1.45	1.33 ± 1.08	**< 0.001**
Vitamin D (IU)	30.89 ± 10.3	27.19 ± 9.09	30.08 ± 9.60	32.30 ± 10.04	33.98 ± 10.05	**< 0.001**
Uric acid (mg/dl)	4.86 ± 1.19	5.04 ± 1.22	4.87 ± 1.21	4.78 ± 1.20	4.67 ± 1.10	**< 0.001**
Type 2 diabetes Mellitus, n (%)	354 (7.0)	136 (10.8)	96 (7.6)	70 (5.5)	52 (4.1)	**< 0.001**
Hypertension, n (%)	912 (18.0)	296 (23.4)	220 (17.4)	194 (15.3)	202 (16.0)	**< 0.001**
Dietary index for gut microbiota score	6.92 ± 1.92	4.55 ± 1.26	6.31 ± 0.39	7.62 ± 0.37	9.20 ± 1.10	**< 0.001**

*Abbreviations*: *WSI* Wealth Score Index, *SBP* Systolic blood pressure, *DBP* Diastolic blood pressure, *HOMA-IR* Homeostatic Model Assessment for Insulin Resistance, *LDL* Low Density Lipoprotein cholesterol, *HDL* High Density Lipoprotein cholesterol, *ALT* Alanine Amino transferase, *AST* Aspartate Amino transferase, *GGT* Gamma-glutamyl transferase, *CRP* C-reactive protein, *CAP* Controlled attenuation parameter

^a^Data are presented as mean ± standard deviation for continuous variables and number (percent) for non-continuous variables

^b^*P*-value was obtained from linear regression analysis for continuous variable, and χ2 test for categorical variables, respectively, according to the category of dietary index for gut microbiota

With respect to anthropometric measures, individuals in Q4 had significantly lower body weight, BMI, WC, and FMI compared to those in Q1 (all P-trend < 0.001). Similarly, cardiometabolic risk factors improved across increasing quartiles of DI-GM, with significant reductions observed in SBP, and DBP, FBS, fasting insulin levels, and HOMA-IR (all P-trend < 0.001).

Regarding lipid profile, participants in the highest quartile had significantly lower levels of TG, TC, and LDL-C, along with higher levels of HDL-C (all P-trend < 0.001). Liver enzymes, including ALT, AST, and GGT, also showed a decreasing trend across quartiles (all P-trend < 0.001).

In addition, markers of liver fat and inflammation demonstrated favorable patterns across DI-GM quartiles. CAP and CRP levels decreased significantly from Q1 to Q4 (P-trend < 0.001). Conversely, serum vitamin D levels increased, while uric acid levels decreased across quartiles (both P-trend < 0.001). Furthermore, the prevalence of type 2 diabetes mellitus and hypertension decreased significantly with higher adherence to DI-GM (P-trend < 0.001 for both).

### Dietary intake according to DI-GM score

Table 2 presents the dietary intake of participants across quartiles of the DI-GM. No significant differences were observed in total energy intake or the percentage of energy derived from carbohydrates across DI-GM quartiles (P-trend = 0.48 and 0.31, respectively). However, the percentage of energy derived from protein increased, while the contribution from total fat, trans-fatty acids, and saturated fatty acids decreased significantly across quartiles (all P-trend < 0.001).

**Table 2:** Dietary intake of participants across quartiles of dietary index for gut microbiota^a^

	Q1 (*n* = 1264)	Q2 (*n* = 1264)	Q3 (*n* = 1262)	Q4 (*n* = 1266)	*P*-trend^b^
Total energy intake (Kcal)	2212.04 ± 374.43	2206.90 ± 344.36	2214.09 ± 336.46	2219.75 ± 319.80	0.48
Carbohydrate intake (% of energy)	60.86 ± 3.77	60.65 ± 3.61	60.82 ± 3.43	60.95 ± 3.53	0.31
Protein intake (% of energy)	12.90 ± 1.40	13.29 ± 1.44	13.68 ± 1.30	13.74 ± 1.13	**< 0.001**
Fat intake (% of energy)	28.19 ± 4.17	28.14 ± 3.92	27.63 ± 3.65	27.55 ± 3.70	**< 0.001**
Whole grain (gram/day)	15.20 ± 8.99	18.75 ± 9.60	22.46 ± 9.93	25.61 ± 9.69	**< 0.001**
Refined grain (gram/day)	394.65 ± 78.24	379.39 ± 74.19	373.86 ± 69.99	365.35 ± 66.76	**< 0.001**
Legumes (gram/day)	50.78 ± 16.55	61.78 ± 18.37	70.14 ± 17.49	76.31 ± 17.21	**< 0.001**
Red meat (gram/day)	18.82 ± 8.65	17.06 ± 8.80	16.37 ± 8.71	14.86 ± 8.12	**< 0.001**
Processed meat (gram/day)	10.42 ± 6.95	8.45 ± 6.32	7.51 ± 5.89	5.96 ± 5.29	**< 0.001**
White meat (gram/day)	38.49 ± 14.13	42.96 ± 14.66	45.79 ± 15.00	46.34 ± 15.5	**< 0.001**
Dairy (gram/day)	156.67 ± 30.95	159.35 ± 32.23	165.85 ± 32.90	169.19 ± 34.37	**< 0.001**
Fruits (gram/day)	327.05 ± 60.17	348.00 ± 63.26	366.89 ± 60.24	379.13 ± 5.47	**< 0.001**
Vegetables (gram/day)	465.41 ± 94.91	499.94 ± 105.15	520.27 ± 95.40	546.07 ± 93.51	**< 0.001**
Nuts (gram/day)	4.59 ± 3.81	5.08 ± 3.93	5.72 ± 4.40	5.59 ± 4.21	**< 0.001**
Sugar (gram/day)	46.67 ± 24.77	43.69 ± 23.45	41.48 ± 22.71	41.10 ± 21.72	**< 0.001**
Salt (gram/day)	3.53 ± 2.11	2.99 ± 1.98	2.64 ± 1.80	2.55 ± 1.74	**< 0.001**
Total fiber (gram/day)	19.68 ± 2.43	21.28 ± 2.60	22.43 ± 2.37	23.43 ± 2.17	**< 0.001**
Trans-fatty acid (% of energy)	0.27 ± 0.13	0.23 ± 0.12	0.20 ± 0.11	0.19 ± 0.11	**< 0.001**
Saturated fatty acid (% of energy)	10.32 ± 1.89	10.36 ± 1.77	10.13 ± 1.65	10.15 ± 1.57	**< 0.001**

^a^Data are presented as mean ± standard deviation

^b^*P*-value was obtained from linear regression analysis according to the category of dietary index for gut microbiota

Participants in the highest quartile had a significantly higher intake of whole grains, legumes, white meat, dairy products, fruits, vegetables, and nuts compared with those in the lowest quartile (all P-trend < 0.001). In addition, total dietary fiber intake increased progressively across quartiles. Conversely, consumption of refined grains, red meat, processed meat, sugar, and salt decreased significantly with increasing DI-GM score (all P-trend < 0.001).

### Association between DI-GM and MASLD

The association between DI-GM and odds of MASLD is presented in Table 3. In the crude model, higher adherence to DI-GM was strongly and inversely associated with the odds of MASLD. Compared with participants in the lowest quartile, the ORs decreased progressively across higher quartiles, reaching 0.21 (95% CI: 0.16–0.28; P for trend < 0.001) in Q4. This inverse association remained significant after adjustment for potential confounders. In the fully adjusted model (Model 3), participants in the highest quartile had 42% lower odds of MASLD compared with those in the lowest quartile (OR: 0.58; 95% CI: 0.42–0.80; P for trend < 0.01). When DI-GM was analyzed as a continuous variable, each standard deviation increase in the score was associated with a significantly lower odds of MASLD across all models, including the fully adjusted model (OR: 0.72; 95% CI: 0.65–0.81; *P* < 0.001).

**Table 3:** Odds ratios (95% confidence intervals) for MASLD incidence according to the proportion of dietary index for gut microbiota (modified 12-item index)

	Q1	Q2	Q3	Q4	*P*-trend	Continues (Per SD increment)	*P*-value
Median Score	4.6	6.3	7.5	9.2			
Cases/populations	248/1264	157/1264	94/1264	63/1266		562/5058	
Crude model	1.00 (Ref)	**0.58 (0.46–0.72)**	**0.33 (0.25–0.42)**	**0.21 (0.16–0.28)**	**< 0.001**	**0.49 (0.44–0.54)**	**< 0.001**
Model 1^a^	1.00 (Ref)	**0.59 (0.47–0.74)**	**0.35 (0.27–0.45)**	**0.23 (0.17–0.30)**	**< 0.001**	**0.50 (0.46–0.55)**	**< 0.001**
Model 2^b^	1.00 (Ref)	0.82 (0.64–1.05)	**0.70 (0.53–0.94)**	**0.52 (0.38–0.72)**	**< 0.001**	**0.69 (0.62–0.77)**	**< 0.001**
Model 3^c^	1.00 (Ref)	0.78 (0.61–1.01)	0.75 (0.56–1.01)	**0.58 (0.42–0.80)**	**< 0.01**	**0.72 (0.65–0.81)**	**< 0.001**

*MASLD* Metabolic dysfunction-associated steatotic liver disease

Significant *p*-values are highlighted in bold

^a^Model 1 adjusted for age and sex

^b^Model 2 additionally adjusted for Model 1 and fat mass index, physical activity, wealth score index, education level, smoking, sleep disorders, hypertension, and type 2 diabetes mellitus

^c^Model 3 additionally adjusted for total energy intake

To further evaluate the robustness of these findings and address potential concerns regarding the modified 12-item index, additional analyses were conducted using the original 14-item DI-GM (including imputed avocado and green tea) (Supplementary Table 2). The results were largely consistent, demonstrating a similar inverse association between DI-GM and MASLD. In the fully adjusted model, participants in the highest quartile had significantly lower odds of MASLD compared with those in the lowest quartile (OR: 0.58; 95% CI: 0.41–0.80; P for trend < 0.01). Likewise, each standard deviation increase in the 14-item score was associated with reduced odds of MASLD (OR: 0.90; 95% CI: 0.86–0.95; *P* < 0.001).

**Fig. 2:**
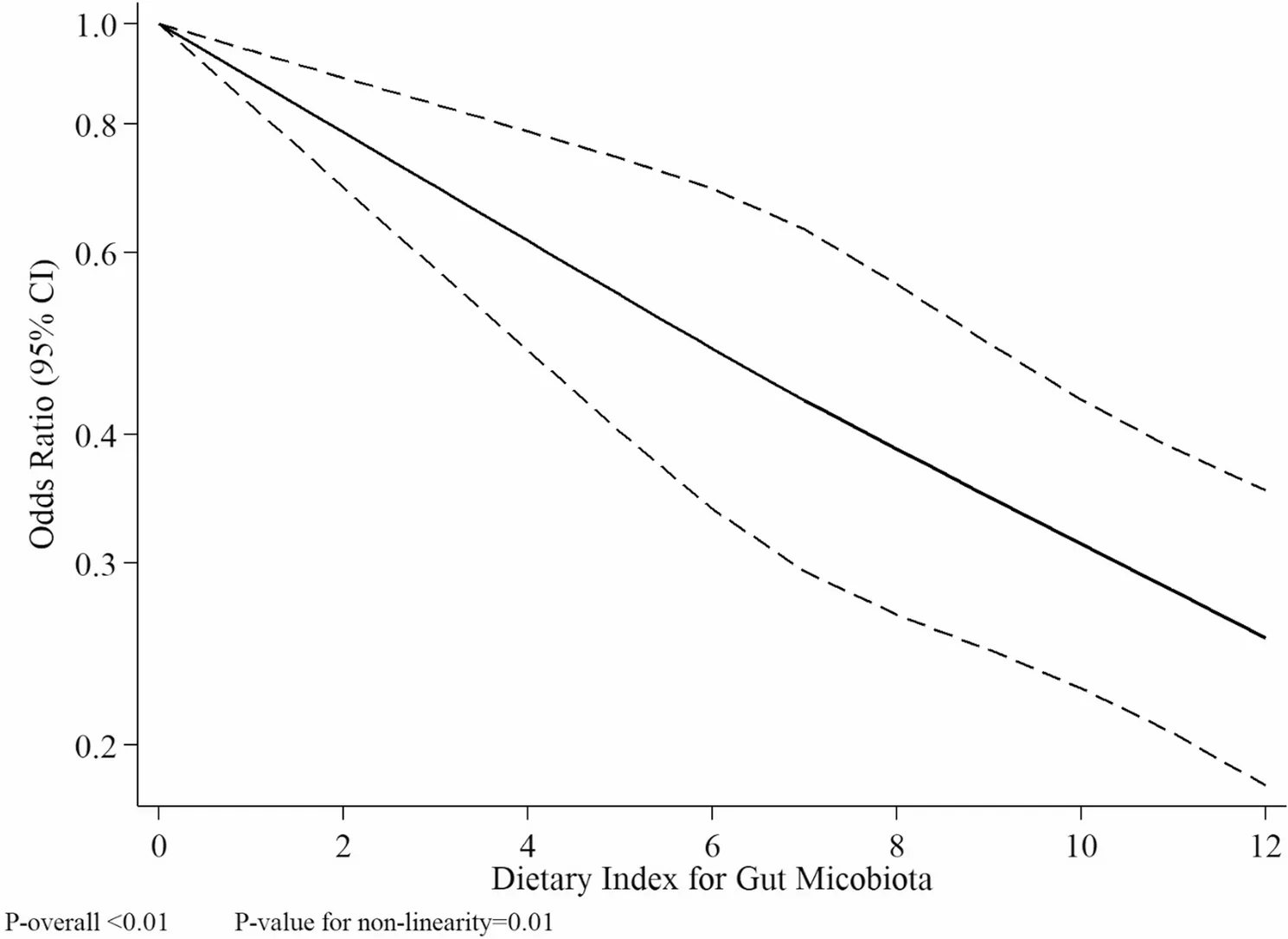
Multivariable adjusted spline curve for the association between dietary index for gut microbiota score and the odds of metabolic dysfunction-associated steatosis liver disease. The model was adjusted for age, sex, fat mass index, physical activity, wealth score index, education level, smoking status, sleep disorders, type 2 diabetes mellitus, hypertension, and total energy intake. The solid line represents the adjusted odds ratio, and the shaded area indicates the 95% confidence interval (CI). The median DI-GM score was used as the reference value

Figure 2 shows the multivariable-adjusted spline curve for the association between DI-GM and the odds of MASLD. The results indicate a significant inverse dose–response relationship (P-overall < 0.01), suggesting that higher DI-GM scores are associated with lower odds of MASLD. Moreover, there is evidence of a non-linear association (P for non-linearity = 0.01), indicating that the rate of risk reduction is not constant across the range of DI-GM scores.

Subgroup analyses examining the association between the DI-GM and the odds of MASLD are presented in Table 4. Overall, the inverse association between the DI-GM and MASLD appeared more pronounced in several population subgroups. A significant interaction was observed by age (P for interaction < 0.01). Among participants older than 45 years, higher adherence to the DI-GM was associated with substantially lower odds of MASLD, with individuals in the highest quartile exhibiting a 59% lower odds compared with those in the lowest quartile (OR: 0.41; 95% CI: 0.25–0.68; P-trend = 0.01). In contrast, no clear association was observed among participants younger than 45 years. Sex also significantly modified the association (P for interaction = 0.01). Among women, higher scores of the DI-GM were consistently associated with lower odds of MASLD across quartiles, with a significant trend (P-trend < 0.001). Women in the highest quartile had 54% lower odds of MASLD compared with those in the lowest quartile (OR: 0.46; 95% CI: 0.28–0.76). In men, however, no statistically significant association was observed. When stratified by BMI, an inverse association was observed in both BMI categories, although the interaction was not statistically significant (P for interaction = 0.31). Participants with BMI < 25 kg/m² in the highest quartile had 62% lower odds of MASLD (OR: 0.38; 95% CI: 0.17–0.85; P-trend = 0.01), whereas those with BMI ≥ 25 kg/m² showed a more modest reduction (OR: 0.66; 95% CI: 0.46–0.96; P-trend = 0.03). Stratified analyses by smoking status showed that the inverse association was evident among non-smokers (P-trend = 0.01), with individuals in the highest quartile having 44% lower odds of MASLD (OR: 0.56; 95% CI: 0.39–0.79), whereas no significant association was observed among smokers (P for interaction = 0.56). Similarly, the association was apparent among participants without hypertension (P-trend = 0.01) but not among those with hypertension (P-trend = 0.92), although the interaction was not statistically significant (P for interaction = 0.95). A comparable pattern was observed for type 2 diabetes mellitus, with significant inverse associations among participants without diabetes (P-trend = 0.01) but not among those with diabetes (P-trend = 0.70), without evidence of interaction (P for interaction = 0.28). Stratification by WSI indicated that the inverse association was stronger among individuals with WSI below the median (P-trend = 0.01), whereas no statistically significant trend was observed among those above the median; however, the interaction was not significant (P for interaction = 0.65). Finally, participants with lower physical activity levels demonstrated a significant inverse association (P-trend = 0.01), whereas the association was not significant among those with higher physical activity (P-trend = 0.27), with no evidence of interaction (P for interaction = 0.12).

**Table 4:** Subgroup analysis of the association between dietary index for gut microbiota and the odds of MASLD^a^

	Q1	Q2	Q3	Q4	*P*-trend	*P* for interaction
Age						
< 45 years old	1.00 (Ref)	0.79 (0.56–1.12)	0.73 (0.49–1.10)	0.77 (0.50–1.18)	0.13	**< 0.01**
> 45years old	1.00 (Ref)	0.74 (0.54–1.12)	0.78 (0.51–1.19)	**0.41 (0.25–0.68)**	**0.01**	
Sex						
Men	1.00 (Ref)	1.06 (0.73–1.54)	0.95 (0.64–1.41)	0.76 (0.48–1.18)	0.25	**0.01**
Women	1.00 (Ref)	**0.62 (44-0.87)**	**0.63 (0.41–0.98)**	**0.46 (0.28–0.76)**	**< 0.001**	
Body mass index						
< 25 kg/m2	1.00 (Ref)	0.85 90.46–1.58)	0.61 (0.31–1.19)	**0.38 (0.17–0.85)**	**0.01**	0.31
> 25 kg/m2	1.00 (Ref)	0.77 (0.59–1.02)	0.83 (0.60–1.15)	**0.66 (0.46–0.96)**	**0.03**	
Smoking						
Yes	1.00 (Ref)	1.36 (0.67–2.76)	0.82 (0.32–2.09)	0.64 (0.26–1.59)	0.28	0.56
No	1.00 (Ref)	**0.71 (0.55–0.94)**	0.74 (0.54-1.00)	**0.56 (0.39–0.79)**	**0.01**	
Hypertension						
Yes	1.00 (Ref)	0.81 (0.46–1.40)	1.03 (0.56–1.89)	0.94 (0.49–1.79)	0.92	0.95
No	1.00 (Ref)	0.76 (0.57–1.01)	**0.68 (0.49–0.96)**	**0.49 (0.33–0.71)**	**0.01**	
Type 2 diabetes mellitus						
Yes	1.00 (Ref)	1.77 (0.94–3.34)	1.59 (0.77–3.28)	0.89 (0.36–2.19)	0.70	0.28
No	1.00 (Ref)	**0.67 (0.51–0.88)**	**0.66 (0.48–0.90)**	**0.53 (0.37–0.75)**	**0.01**	
Wealth score index						
< Median (68.86)	1.00 (Ref)	**0.68 (0.50–0.94)**	0.68 (0.46-1.00)	**0.52 (0.34–0.79)**	**0.01**	0.65
> Median (68.86)	1.00 (Ref)	1.00 (0.66–1.51)	0.87 (0.56–1.37)	0.68 (0.40-1.167)	0.17	
Physical activity						
< Median (23.74)	1.00 (Ref)	0.78 (0.58–1.04)	0.81 (0.58–1.13)	**0.56 (0.38–0.82)**	**0.01**	0.12
> Median (23.74)	1.00 (Ref)	0.87 (0.52–1.45)	0.71 (0.39–1.28)	0.77 (0.41–1.42)	0.27	

*MASLD* Metabolic dysfunction-associated steatotic liver disease

^a^Multivariable logistic regression was used to estimate odds ratios (ORs) and 95% confidence intervals (CIs). Models were adjusted for age, sex, physical activity, wealth score index, education level, smoking, fat mass index, sleep disorders, hypertension, type 2 diabetes mellitus, and total energy intake. For subgroup analyses, the stratifying variable was included as a main effect with a product (interaction) term, and the corresponding continuous covariate was omitted from the adjustment set for that subgroup to avoid over-adjustment (e.g., age was not included when stratifying by age)

The robustness of the association between the DI-GM and the odds of MASLD was further evaluated through multiple sensitivity analyses (Table 5). After excluding participants with > 10% weight gain from baseline, the inverse association remained significant, with participants in the highest quartile showing lower odds of MASLD compared to the lowest quartile (OR: 0.61; 95% CI: 0.40–0.94; P for trend = 0.01). Similarly, the association persisted after additional adjustment for the triglyceride-to-HDL ratio, serum uric acid, dietary saturated fatty acids, and dietary sugar (all P for trend ≤ 0.01). However, the association was attenuated and became non-significant after further adjustment for baseline CAP, HOMA-IR, and serum vitamin D levels. Adjustment for CRP also weakened the association, although a modest inverse relationship remained when DI-GM was analyzed as a continuous variable (*P* = 0.04). Notably, additional adjustment for dietary fiber substantially attenuated the association across quartiles, suggesting that fiber intake may play a key role in the observed relationship. Overall, these findings indicate that the inverse association between DI-GM and MASLD is robust to multiple adjustments, but may be partially mediated or confounded by metabolic factors, liver fat accumulation, and dietary components, particularly fiber.

**Table 5:** Sensitivity analysis for developing MASLD according to the proportion of planetary health diet index for gut microbiota^a^

	Q1	Q2	Q3	Q4	*P*-trend	Continues (Per SD increment)	*P*-value
Excluding participants with weight gain of > 10% from baseline	1.00 (Ref)	0.73 (0.52–1.01)	0.72 (0.48–1.06)	0.61 (0.40–0.94)	0.01	0.73 (0.64–0.85)	< 0.001
Additional adjusted for baseline CAP score	1.00 (Ref)	1.02 (0.78–1.34)	1.05 (0.77–1.43)	1.31 (0.91–1.87)	0.21	0.93 (0.83–1.05)	0.27
Additional adjusted for baseline HOMA-IR	1.00 (Ref)	0.91 (0.68–1.19)	1.17 (0.85–1.61)	1.38 (0.96–1.98)	0.08	0.94 (0.83–1.06)	0.35
Additional adjusted for baseline TG to HDL ratio	1.00 (Ref)	0.77 (0.60-1.00)	0.78 (0.58–1.05)	**0.61 (0.44–0.84)**	**0.01**	**0.74 (0.66–0.82)**	**< 0.001**
Additional adjusted for baseline C-reactive protein	1.00 (Ref)	1.00 (0.76–1.32)	1.03 (0.75–1.43)	1.09 (0.76–1.53)	0.64	**0.88 (0.78–0.99)**	**0.04**
Additional adjusted for baseline serum vitamin D	1.00 (Ref)	0.87 (0.67–1.12)	0.85 (0.63–1.14)	0.74 (0.53–1.04)	0.07	1.08 (0.95–1.23)	0.20
Additional adjusted for baseline serum uric acid	1.00 (Ref)	0.78 (0.61–1.01)	0.76 (0.57–1.02)	**0.61 (0.43–0.83)**	**0.01**	**0.73 (0.66–0.81)**	**< 0.001**
Additional adjusted for dietary saturated fatty acid	1.00 (Ref)	0.79 (0.61–1.1)	0.76 (0.57–1.02)	**0.58 (0.42–0.80)**	**< 0.001**	**0.73 (0.65–0.81)**	**< 0.001**
Additional adjusted for dietary fiber	1.00 (Ref)	**0.53 (0.3–0.77)**	**0.56 (0.38–0.82)**	0.80 (0.54–1.18)	0.01	**0.84 (0.79–0.90)**	**< 0.001**
Additional adjusted for dietary sugar	1.00 (Ref)	0.79 (0.62–1.02)	0.79 (0.59–1.06)	0.62 (0.42–0.87)	< 0.001	**0.74 (0.67–0.83)**	**< 0.001**

*MASLD* Metabolic dysfunction-associated steatotic liver disease

Significant *p*-values are highlighted in bold

^a^Multivariable logistic regression was used to estimate odds ratios (ORs) and 95% confidence intervals (CIs). Models were adjusted for age, sex, physical activity, wealth score index, education level, smoking, fat mass index, sleep disorders, hypertension, type 2 diabetes mellitus, and total energy intake

The potential mediating roles of selected biochemical variables in the association between the dietary index for gut microbiota and the odds of MASLD are presented in Table 6. Mediation analyses indicated that several metabolic and liver-related factors substantially contributed to the observed association. CAP showed a strong statistical mediation signal; however, because hepatic steatosis assessed by CAP contributes to the definition of MASLD, these findings should be interpreted cautiously and considered exploratory. The indirect effect through CAP was statistically significant (estimate: −0.01473; 95% CI: −0.01812, − 0.01194; *P* < 0.001), accounting for 83.5% of the total association. In contrast, the direct effect was not statistically significant (estimate: −0.00289; 95% CI: −0.00888, 0.00189; *P* = 0.26), suggesting that a large proportion of the association between the dietary index and MASLD operates through liver steatosis as reflected by CAP. Similarly, insulin resistance assessed by HOMA-IR showed a substantial mediating role. The indirect effect was significant (estimate: −0.01579; 95% CI: −0.01954, − 0.01284; *P* < 0.001), explaining 90.8% of the total effect. The direct effect was not statistically significant (estimate: −0.00158; 95% CI: −0.00794, 0.00347; *P* = 0.56), indicating that the association between the dietary index and MASLD may largely operate through pathways related to insulin resistance. Vitamin D also partially mediated the association. Both the direct (estimate: −0.01324; 95% CI: −0.02191, − 0.00634; *P* < 0.001) and indirect effects (estimate: −0.00619; 95% CI: −0.00818, − 0.00451; *P* < 0.001) were statistically significant, with 31.8% of the total effect explained by the mediation pathway. These findings suggest that vitamin D may contribute to the association between the dietary index and MASLD, although a substantial portion of the effect remains independent of this mediator.

**Table 6:** The mediation effect of biochemical and dietary variables on the association between dietary index for gut microbiota and the odds of MASLD^a^

Mediator		Direct effect	Indirect effect (Mediated)	Total effect	Proportion mediated (%)
Controlled attenuation parameter	Estimate	-0.00289	-0.01473	-0.01763	83.5
	95% CI	(-0.00888, 0.00189)	(-0.01812, -0.01194)	(-0.02576, -0.01101)	
	P-value	0.26	**< 0.001**	**< 0.001**	**< 0.001**
HOMA-IR	Estimate	-0.00158	-0.01579	-0.01738	90.8
	95% CI	(-0.00794, 0.00347)	(-0.01954, -0.01284)	(-0.02600, -0.01051)	
	P-value	0.56	**< 0.001**	**< 0.001**	**< 0.001**
Vitamin D	Estimate	-0.01324	-0.00619	-0.01944	31.8
	95% CI	(-0.02191, -0.00634)	(-0.00818, -0.00451)	(-0.02888, -0.01116)	
	P-value	< 0.001	**< 0.001**	**< 0.001**	**< 0.001**

^a^Models were adjusted for age, sex, physical activity, wealth score index, education level, smoking, fat mass index, sleep disorders, hypertension, type 2 diabetes mellitus, and total energy intake

## Discussion

In this large-scale prospective cohort study of 5,058 Iranian adults, we observed a significant inverse association between adherence to DI-GM and five-year incidence of MASLD. Participants in the highest quartile of DI-GM scores demonstrated 42% lower odds of developing MASLD compared to those in the lowest quartile, after comprehensive adjustment for confounders. A non-linear dose-response relationship was evident, with steeper risk reduction at lower index scores that plateaued at higher adherence levels. Notably, the protective association varied across population subgroups and was substantially mediated through metabolic pathways involving hepatic steatosis, insulin resistance, and vitamin D status.

### Consistency with existing evidence and advancement of knowledge

Our findings align with and extend previous cross-sectional investigations examining DI-GM and fatty liver disease. Multiple NHANES-based studies have consistently reported inverse associations between DI-GM scores and NAFLD or MAFLD prevalence, with odds reductions ranging from 35% to 58% when comparing highest to lowest adherence categories [10–14]. However, these studies were limited by their cross-sectional design, precluding temporal inference. Our prospective approach addresses this critical gap, demonstrating that higher DI-GM scores precede and predict lower MASLD incidence over five years, thereby strengthening evidence for a potential protective role of gut microbiota-targeted dietary patterns.

The magnitude of association observed in our study (OR: 0.58 for Q4 vs. Q1) is comparable to previous reports, yet our continuous analysis revealed that each standard deviation increase in DI-GM score conferred 28% lower odds of MASLD. This dose-response relationship, confirmed through restricted cubic spline analysis showing significant non-linearity (P-nonlinearity = 0.01), suggests threshold effects whereby moderate improvements in diet quality may yield substantial metabolic benefits, with diminishing marginal returns at very high adherence levels.

### Subgroup heterogeneity and effect modification

Our subgroup analyses revealed important effect modification by demographic and metabolic characteristics, providing insights into populations who may derive greatest benefit from dietary interventions targeting gut microbiota. The protective association was significantly stronger among women (Q4 vs. Q1: OR = 0.46) compared to men (OR = 0.73), and among individuals aged over 45 years (OR = 0.41) versus younger adults (OR = 0.76), with significant interaction terms for both sex and age. These findings diverge from several NHANES-based studies that reported no significant subgroup differences [10, 12], though one study similarly identified stronger associations among women [11].

The sex-specific difference may reflect hormonal influences on gut microbiota composition and hepatic lipid metabolism. Estrogen has been shown to modulate bile acid metabolism and gut microbial diversity, potentially enhancing responsiveness to dietary interventions [28].

The age-related effect modification suggests that middle-aged and older adults, who face higher baseline MASLD risk due to age-related metabolic decline, may experience greater absolute risk reduction from dietary improvements.

Importantly, the protective association remained significant only among participants without baseline hypertension or type 2 diabetes, and among those with lower wealth scores. This pattern suggests that dietary interventions may be most effective in earlier stages of metabolic dysfunction, before establishment of overt cardiometabolic disease. The stronger association among lower socioeconomic groups, while requiring cautious interpretation, may indicate greater potential for dietary modification to address health disparities, though access barriers to recommended foods must be considered in intervention design.

### Mediation through metabolic pathways

Our mediation analyses provide novel insights into potential mechanisms linking dietary patterns to MASLD risk. Exploratory mediation analyses suggested substantial statistical mediation through CAP score (83.5% mediated), HOMA-IR (90.8% mediated), and vitamin D levels (31.8% mediated). However, these findings should be interpreted cautiously because the assumptions required for causal mediation may not be fully satisfied in the present observational setting. These findings are consistent with the hypothesis that the association between DI-GM and MASLD may operate, at least in part, through pathways involving hepatic steatosis and insulin resistance, with a smaller contribution from vitamin D-related mechanisms.

The observed statistical mediation by CAP and HOMA-IR is consistent with the hypothesis that hepatic steatosis and insulin resistance may represent important biological pathways linking dietary patterns to MASLD risk. However, these findings do not establish causal mediation and should be regarded as hypothesis-generating. This is consistent with one previous study reporting BMI as a 59% mediator [11], though our use of direct steatosis measurement (CAP) rather than adiposity provides additional exploratory insight into potential biological pathways, although interpretation is limited by the conceptual overlap between CAP and the outcome definition. The vitamin D mediation, while modest, aligns with emerging evidence linking vitamin D status to both gut microbiota composition and hepatic inflammation [29].

These mediation patterns were further supported by our sensitivity analyses, where adjustment for baseline CAP or HOMA-IR substantially attenuated the DI-GM-MASLD association, supporting the possibility that these variables may represent important intermediate pathways.

### Distinguishing observed associations from mechanistic inference

It is critical to emphasize that our study did not include direct measurements of gut microbiota composition, microbial metabolites, or gut barrier function. Therefore, while we can document associations between dietary patterns and MASLD incidence, we cannot directly demonstrate that these associations operate through microbiota-mediated mechanisms. The mechanistic pathways discussed below are inferred from existing literature and represent plausible biological explanations that require validation in future studies incorporating microbiome profiling.

The theoretical framework linking diet, gut microbiota, and liver health is supported by substantial experimental and observational evidence from other populations. Dietary fibers serve as fermentable substrates for gut bacteria, promoting production of short-chain fatty acids (SCFAs) including butyrate, acetate, and propionate [30–33]. These metabolites may enhance intestinal barrier integrity, modulate inflammatory signaling, and influence hepatic lipid and glucose metabolism [31–33]. Soluble fibers specifically promote growth of beneficial bacterial genera such as Faecalibacterium and Roseburia [32, 34], while insoluble fibers support gut transit and fecal bulk [32, 35].

Beyond fiber, other DI-GM components may influence the gut-liver axis through distinct mechanisms described in prior literature. Polyphenol-rich foods (fruits, vegetables, nuts) may modulate microbial composition and reduce oxidative stress [36]. Fermented dairy products provide probiotics that may compete with pathogenic bacteria [37]. Conversely, high intake of red meat, processed foods, and added sugars has been associated with pro-inflammatory microbial profiles and increased intestinal permeability in experimental studies [38, 39].

Proposed mechanisms linking gut dysbiosis to MASLD include: increased translocation of bacterial lipopolysaccharides triggering hepatic inflammation via Toll-like receptor 4 signaling [40, 41]; altered bile acid metabolism affecting farnesoid X receptor signaling and lipid homeostasis [42, 43]; bacterial production of endogenous ethanol contributing to oxidative stress [30, 43]; and conversion of choline to trimethylamine-N-oxide, reducing hepatic lipid export capacity [44, 45]. However, we emphasize that these represent hypothesized pathways based on external evidence, not mechanisms directly evaluated in our cohort.

### Limitations requiring acknowledgment

Several limitations warrant consideration. First and most importantly, our study lacks direct microbiome measurements. While the DI-GM was developed and validated against fecal microbiota diversity in other populations [18], we cannot confirm whether dietary patterns in our cohort actually modified gut microbial composition, nor whether such modifications mediated the observed associations with MASLD. The mechanistic interpretations presented above, while biologically plausible and supported by literature, remain speculative in the context of our data. Future studies incorporating paired dietary, microbiome, and metabolic assessments are essential to validate these proposed pathways. Second, dietary intake was assessed using a food frequency questionnaire, introducing potential recall bias and measurement error. Although we employed a validated FFQ adapted for Iranian dietary patterns and trained interviewers, systematic misreporting cannot be excluded. Because dietary misclassification arising from FFQ measurement error is likely to be largely non-differential with respect to future MASLD status, it may have attenuated the observed associations toward the null, potentially leading to an underestimation of the true effect size. Third, the DI-GM was calculated using 12 of 14 original components due to unavailable data on green tea and avocado consumption. Sensitivity analyses using an alternative 14-item DI-GM score constructed under prespecified assumptions regarding avocado and green tea consumption yielded results consistent with those of the primary analyses, suggesting that the omission of these components had minimal impact on our conclusions. It should be noted that neither the original 14-item DI-GM nor the adapted 12-item version used in the present study has been formally validated in an Iranian population, and caution is therefore warranted in assuming equivalent construct validity and predictive performance to the populations in which the index was originally developed. Moreover, avocado and green tea consumption were not assessed in the study FFQ and therefore represented structurally unavailable components rather than conventionally missing data. Consequently, the alternative 14-item DI-GM score relied on assumptions regarding the distribution of these dietary components within the study population and should be interpreted cautiously. Although sensitivity analyses yielded results comparable to those of the primary 12-item score, some degree of exposure misclassification and bias in the estimation of DI-GM adherence cannot be excluded. Fourth, hepatic steatosis was defined using a CAP threshold of ≥ 238 dB/m. Although this cut‑off has been suggested as a sensitive threshold for detecting the presence of steatosis, other studies have applied higher CAP thresholds (e.g., ≥ 274 or ≥ 285 dB/m) [10, 12], and several previous DI‑GM studies have assessed hepatic steatosis using surrogate indices such as the Fatty Liver Index (FLI) [11, 13, 14]. Differences in diagnostic criteria may influence the estimated prevalence of MASLD and may limit direct comparability with findings from studies using alternative definitions. Fifth, although we adjusted for an extensive set of potential confounders, residual confounding due to unmeasured or imperfectly measured factors, including genetic predisposition, environmental exposures, and unmeasured lifestyle characteristics, cannot be excluded and may have influenced the observed estimates. Finally, participants were recruited from individuals attending a comprehensive health center using a convenience‑based approach. Individuals who seek care at health centers may differ from the general population in terms of health awareness, baseline health status, or socioeconomic characteristics. In addition, the exclusion of participants with pre‑existing conditions further narrowed the analytic sample. Therefore, the findings may not be fully generalizable to the broader population and may be most applicable to health‑seeking urban Iranian adults. Caution is also warranted when extrapolating these results to populations with substantially different dietary habits, microbiome profiles, or genetic backgrounds. Future studies using more representative population‑based samples are needed to confirm these findings. The convenience-based recruitment strategy and exclusion of participants with pre-existing diseases may also have introduced selection bias, potentially resulting in a healthier and more health-conscious study population than the general population. Such individuals may be more likely to adhere to healthier dietary patterns and engage in other health-promoting behaviors that reduce MASLD risk. Consequently, the observed inverse association between DI-GM and MASLD may have been overestimated because residual healthy-user characteristics could not be fully accounted for. Conversely, the restricted variability in risk profiles among relatively healthy participants may also have attenuated the association. Therefore, the direction and magnitude of this potential bias cannot be determined with certainty but should be considered when interpreting the findings and their generalizability. In addition, some variables included in the sensitivity analyses, particularly CAP and HOMA-IR, may lie on the causal pathway between dietary patterns and MASLD. Consequently, adjustment for these factors may have resulted in partial overadjustment and attenuation of the observed associations. The mediation analyses should therefore be interpreted as hypothesis-generating rather than establishing definitive causal pathways. An additional limitation relates to the use of CAP in both the outcome definition and the exploratory mediation and sensitivity analyses. Because hepatic steatosis assessed by CAP constitutes one component of the MASLD definition, analyses involving CAP may be subject to conceptual overlap and partial circularity. Consequently, the strong statistical mediation observed for CAP and the attenuation of associations after adjustment for CAP should not be interpreted as definitive evidence of causality but rather as exploratory findings intended to generate hypotheses regarding potential biological pathways. An additional limitation concerns the interpretation of the mediation analyses. Causal mediation analysis relies on strong assumptions, including the absence of unmeasured confounding and correct temporal ordering between exposure, mediator, and outcome. These assumptions are difficult to verify and may not be fully met in the present observational setting. Furthermore, the mediators were assessed at baseline and may already reflect subclinical disease processes that preceded the diagnosis of incident MASLD. Therefore, the mediation findings should be interpreted as exploratory and hypothesis-generating rather than providing definitive evidence regarding causal mechanisms.

An additional limitation relates to the analytical approach used to model incident MASLD. Because the exact timing of disease onset was unavailable and outcomes were assessed at a single follow-up examination, we used logistic regression rather than time-to-event models. Consequently, the estimated odds ratios should not be interpreted as direct estimates of risk ratios. Given the non-rare occurrence of MASLD in our cohort (approximately 11% over five years), the estimated odds ratios may somewhat overstate the magnitude of the corresponding relative risks, although the direction and statistical significance of the associations are unlikely to be materially affected.

### Strengths and clinical implications

This study provides the first prospective evidence linking DI-GM to MASLD incidence, addressing a critical limitation of prior cross-sectional research. The large sample size, comprehensive covariate data, and ability to conduct subgroup, sensitivity, and mediation analyses strengthen confidence in our findings. Assessment of MASLD using controlled attenuation parameter provides objective measurement superior to surrogate markers.

From a clinical perspective, the DI-GM may serve as a practical tool for identifying dietary patterns associated with MASLD risk, potentially guiding personalized nutritional counseling. The index emphasizes accessible dietary modifications—increasing fiber-rich plant foods while reducing processed meats and added sugars—that align with existing dietary guidelines. The substantial mediation through hepatic steatosis and insulin resistance suggests that dietary interventions targeting gut microbiota may influence MASLD through established pathogenic pathways.

At the population level, these findings support dietary interventions promoting microbiota-friendly food patterns as cost-effective strategies for MASLD prevention, particularly among women, middle-aged adults, and individuals in earlier stages of metabolic dysfunction. However, interventional trials are needed to confirm whether improving DI-GM adherence causally reduces MASLD incidence and to elucidate the role of gut microbiota in mediating such effects. Furthermore, although subgroup and mediation analyses provided valuable mechanistic and population-specific insights, some subgroup categories included relatively few outcome events, resulting in wider confidence intervals and greater statistical uncertainty. Therefore, these findings should be interpreted cautiously and considered exploratory.

## Conclusions

Higher adherence to a dietary pattern theoretically supportive of gut microbiota health, as measured by the DI-GM, was prospectively associated with reduced MASLD incidence in Iranian adults. This association demonstrated a non-linear dose-response relationship, varied across demographic and metabolic subgroups, and was substantially mediated through hepatic steatosis and insulin resistance. While these findings support the potential value of gut microbiota-targeted dietary interventions for MASLD prevention, direct evidence linking dietary patterns to microbiome changes and subsequent metabolic outcomes in this population is needed. Future research incorporating comprehensive microbiome profiling alongside dietary and metabolic assessments will be essential to validate proposed mechanisms and inform evidence-based dietary recommendations for MASLD prevention.

## Supplementary Information


Supplementary Material 1.


## Data Availability

The datasets analyzed in the current study are available from the corresponding author on reasonable request.

## Abbreviations

MASLD: Metabolic dysfunction-associated steatotic liver disease
DI-GM: Dietary Index for Gut Microbiota
FFQ: Food Frequency Questionnaire
OR: Odds ratio
CI: Confidence interval
CAP: Controlled Attenuation Parameter
BMI: Body mass index
WC: Waist circumference
SBP: Systolic blood pressure
DBP: Diastolic blood pressure
FBS: Fasting blood sugar
FPG: Fasting plasma glucose
HbA1c: Hemoglobin A1c
HOMA-IR: Homeostasis model assessment of insulin resistance
CRP: C-reactive protein
ALT: Alanine aminotransferase
AST: Aspartate aminotransferase
GGT: Gamma-glutamyl transferase
TG: Triglycerides
TC: Total cholesterol
HDL-C: High-density lipoprotein cholesterol
LDL-C: Low-density lipoprotein cholesterol
T2DM: Type 2 diabetes mellitus
WSI: Wealth score index
MCA: Multiple correspondence analysis
MAQ: Modifiable Activity Questionnaire
MET: Metabolic equivalent task
DXA: Dual-energy X-ray absorptiometry
FMI: Fat mass index
JNC 7: Joint National Committee 7
ADA: American Diabetes Association
RCT: Randomized controlled trial
PA: Physical activity
RCS: Restricted cubic spline
R: R statistical software
STROBE: Strengthening the Reporting of Observational Studies in Epidemiology
NAFLD: Non-alcoholic fatty liver disease
MAFLD: Metabolic dysfunction-associated fatty liver disease
NCDs: Non-communicable diseases
IR: Insulin resistance
WC: Waist circumference
