# Supplementary material for: Adherence to the dietary index for gut microbiota and the 5-year incidence of metabolic dysfunction-associated steatotic liver disease in Iranian adults: a prospective cohort study
Source: BMC Gastroenterol. 2026 Jun 20;26:535. doi: 10.1186/s12876-026-05036-5 (PMC13528170; doi:10.1186/s12876-026-05036-5)
Supplement: Supplementary file 1 [file 12876_2026_5036_MOESM1_ESM.docx]

**Supplementary Table Legend:**

**Supplementary Table 1.** STROBE Checklist.

**Supplementary Table 2.** Odds ratios (95% confidence intervals) for metabolic dysfunction-associated steatotic liver disease incidence according to quartiles of the 14-item dietary index for gut microbiota (including imputed avocado and green tea).

**Supplementary Table 1.** STROBE Checklist.

STROBE Statement—Checklist of items that should be included in reports of ***cohort studies***

|  | **Item No** | **Recommendation** |
| --- | --- | --- |
| **Title and abstract** | Title page, lines 1–2 | (*a*) Indicate the study’s design with a commonly used term in the title or the abstract |
|  | Abstract, lines 34–59 | (*b*) Provide in the abstract an informative and balanced summary of what was done and what was found |
| **Introduction** | | |
| Background/rationale | Introduction, lines 60–85 | Explain the scientific background and rationale for the investigation being reported |
| Objectives | lines 86–89 | State specific objectives, including any prespecified hypotheses |
| **Methods** | | |
| Study design | lines 90–93 | Present key elements of study design early in the paper |
| Setting | lines 90–100 | Describe the setting, locations, and relevant dates, including periods of recruitment, exposure, follow-up, and data collection |
| Participants | lines 95–108 | (*a*) Give the eligibility criteria, and the sources and methods of selection of participants. Describe methods of follow-up |
|  | Not applicable because this was not a matched cohort study | (*b*) For matched studies, give matching criteria and number of exposed and unexposed |
| Variables | lines 109–153 | Clearly define all outcomes, exposures, predictors, potential confounders, and effect modifiers. Give diagnostic criteria, if applicable |
| Data sources/ measurement | lines 109–171 | For each variable of interest, give sources of data and details of methods of assessment (measurement). Describe comparability of assessment methods if there is more than one group |
| Bias | lines 102–104 and 250–254 (comparison of participants lost to follow-up and strategies to minimize bias) | Describe any efforts to address potential sources of bias |
| Study size | Methods, lines 105–108 | Explain how the study size was arrived at |
| Quantitative variables | lines 173–206 | Explain how quantitative variables were handled in the analyses. If applicable, describe which groupings were chosen and why |
| Statistical methods | lines 173–206 | (*a*) Describe all statistical methods, including those used to control for confounding |
|  | lines 202–210 | (*b*) Describe any methods used to examine subgroups and interactions |
|  | lines 102–104 | (*c*) Explain how missing data were addressed |
|  | follow-up rate was 95% and participants lost to follow-up were compared with completers. | (*d*) If applicable, explain how loss to follow-up was addressed |
|  | lines 211–216 | (*e*) Describe any sensitivity analyses |
| **Results** | | |
| Participants | lines 93–108 and Figure 1 | (a) Report numbers of individuals at each stage of study—eg numbers potentially eligible, examined for eligibility, confirmed eligible, included in the study, completing follow-up, and analysed |
|  | Figure 1 | (b) Give reasons for non-participation at each stage |
|  | Figure 1 | (c) Consider use of a flow diagram |
| Descriptive data | lines 255–284; Tables 1 and 2 | (a) Give characteristics of study participants (eg demographic, clinical, social) and information on exposures and potential confounders |
|  | lines 102–104 | (b) Indicate number of participants with missing data for each variable of interest |
|  | lines 97–100 and Abstract | (c) Summarise follow-up time (eg, average and total amount) |
| Outcome data | lines 285–300 and Table 3 | Report numbers of outcome events or summary measures over time |
| Main results | lines 285–300 and Table 3 | (*a*) Give unadjusted estimates and, if applicable, confounder-adjusted estimates and their precision (eg, 95% confidence interval). Make clear which confounders were adjusted for and why they were included |
|  | Table 1; lines 173–181 and 202–205 | (*b*) Report category boundaries when continuous variables were categorized |
|  | Absolute risk estimates were not calculated because the primary analysis was based on odds ratios from logistic regression models | (*c*) If relevant, consider translating estimates of relative risk into absolute risk for a meaningful time period |
| Other analyses | lines 301–380; Tables 4–6 | Report other analyses done—eg analyses of subgroups and interactions, and sensitivity analyses |
| **Discussion** | | |
| Key results | lines 365–380 | Summarise key results with reference to study objectives |
| Limitations | lines 443–539 | Discuss limitations of the study, taking into account sources of potential bias or imprecision. Discuss both direction and magnitude of any potential bias |
| Interpretation | lines 365–559 and Conclusion (lines 550–559) | Give a cautious overall interpretation of results considering objectives, limitations, multiplicity of analyses, results from similar studies, and other relevant evidence |
| Generalisability | lines 488–499 | Discuss the generalisability (external validity) of the study results |
| **Other information** | | |
| Funding | lines 594–599 | Give the source of funding and the role of the funders for the present study and, if applicable, for the original study on which the present article is based |

*Give information separately for exposed and unexposed groups.

**Note:** An Explanation and Elaboration article discusses each checklist item and gives methodological background and published examples of transparent reporting. The STROBE checklist is best used in conjunction with this article (freely available on the Web sites of PLoS Medicine at http://www.plosmedicine.org/, Annals of Internal Medicine at http://www.annals.org/, and Epidemiology at http://www.epidem.com/). Information on the STROBE Initiative is available at http://www.strobe-statement.org.

| **Supplementary Table 1.** Odds ratios (95% confidence intervals) for metabolic dysfunction-associated steatotic liver disease incidence according to quartiles of the 14-item dietary index for gut microbiota (including imputed avocado and green tea). | | | | | | | |
| --- | --- | --- | --- | --- | --- | --- | --- |
|  | Q1 | Q2 | Q3 | Q4 | P-trend | Continues  (Per SD increment) | P-value |
| Median Score | 5.0 | 7.0 | 8.0 | 10.0 |  |  |  |
| Cases/populations | 307/1706 | 115/930 | 85/955 | 57/1467 |  |  |  |
| Crude model | 1.00 (Ref) | **0.64 (0.51-0.81)** | **0.45 (0.35-0.58)** | **0.18 (0.14-0.25)** | **<0.001** | **0.58 (0.53-0.64)** | **<0.001** |
| Model 1^a^ | 1.00 (Ref) | **0.67 (0.53-0.85)** | **0.46 (0.36-0.59)** | **0.20 (0.15-0.26)** | <0.001 | **0.60 (0.54-0.66)** | **<0.001** |
| Model 2^b^ | 1.00 (Ref) | 1.00 (0.77-1.31) | 0.88 (0.66-1.17) | **0.44 (0.32-0.61)** | **<0.01** | **0.86 (0.77-0.95)** | **<0.01** |
| Model 3^c^ | 1.00 (Ref) | 1.12 (0.86-1.46) | 1.09 (0.81-1.47) | **0.58 (0.41-0.80)** | **<0.01** | **0.90 (0.86-0.95)** | **<0.001** |
| ^a^ Model 1 adjusted for age and sex.  ^b^ Model 2 additionally adjusted for Model 1 and fat mass index, physical activity, wealth score index, education level, smoking, sleep disorders, hypertension, and type 2 diabetes mellitus.  ^c^ Model 3 additionally adjusted for total energy intake. | | | | | | | |
